# Detection of Hydroxychloroquine Retinal Toxicity by Automated Perimetry in 60 Rheumatoid Arthritis Patients With Normal Fundoscopic Findings

**DOI:** 10.5539/gjhs.v8n3p59

**Published:** 2015-06-25

**Authors:** Qader Motarjemizadeh, Naser Samadi Aidenloo, Mohammad Abbaszadeh

**Affiliations:** 1 Department of Ophthalmology, Urmia University of Medical Sciences, Urmia, Iran

**Keywords:** hydroxychloroquine, rheumatoid arthritis, perimetry, retinal toxicity

## Abstract

Hydroxychloroquine (HCQ) is an antimalarial drug used extensively in treatment of autoimmune diseases such as rheumatoid arthritis. Retinal toxicity is the most important side effects of this drug. Even after the drug is discontinued, retinal degeneration from HCQ can continue to progress. Consequently, multiple ophthalmic screening tests have been developed to detect early retinopathy. The aim of the current study was to evaluate the value of central 2-10 perimetry method in early detection of retinal toxicity. This prospective cross-sectional investigation was carried out on 60 rheumatoid arthritis patients, who had been receiving HCQ for at least 6 months and still were on their medication (HCQ intake) at the time of enrollment. An ophthalmologist examined participants using direct and indirect ophthalmoscopy. Visual field testing with automated perimetry technique (central 2-10 perimetry with red target) was performed on all included subjects twice in 6 months interval: The first one at the time of enrollment and the second one 6 months later. Males and females did not show any significant difference in terms of age, duration of therapy, daily and cumulative HCQ dose, anterior or posterior segment abnormalities, hypertension, body mass index, and best corrected visual acuity. Anterior segment was abnormal in 9 individuals including 3 subjects with macular pigmentary changes, 4 individuals with cataract and 2 cases with dry eyes. Moreover, 12 subjects had retinal pigmented epithelium (RPE) in their posterior segments. After 6 months, depressive changes appeared in 12 subjects. Additionally, HCQ therapy worsened significantly the perimetric results of 5 (55.6%) patients with abnormal anterior segment. A same trend was observed in perimetric results of 6 (50.0%) subjects with abnormal posterior segments (P=0.009). The daily dose of HCQ (P=0.035) as well as the cumulative dose of hydroxychloroquine (P=0.021) displayed statistically significant associations with perimetric results. Central 2-10 perimetry is a useful method for early detection of HCQ retinal toxicity, but more comprehensive studies, with larger sample size, longer-term follow-up and more precise techniques are mandatory to confirm HCQ retinal toxicity.

## 1. Introduction

### 1.1 Introduce the Problem

Hydroxychloroquine (HCQ) is an anti-malarial drug that has been used for the treatment of rheumatoid arthritis, systemic lupus erythematosus and some other connective tissue diseases. It has been reported that HCQ administration is associated with toxic side effects on the retinal pigment epithelium (RPE) as well as on the outer retina (Finbloom, Silver, Newsome, & Gunkel, 1985; Mavrikakis et al., 2003; Shearer & Dubois, 1967; Weiner, Sandberg, Gaudio, Kini, & Berson, 1991). Early signs of functional disturbances include pigmentary abnormalities of the PRE, paracentral visual field defects, color vision deficiencies and reading difficulties. If these early signs are overlooked, a severe retinal toxicity with irreversible visual loss may develop.

Retinal toxicity from HCQ is of serious ophthalmologic concern. Because even after the drug is discontinued, there is little if any visual recovery. Additionally, it has been shown that the retinal degeneration caused by HCQ can continue to progress (Marmor, Carr, Easterbrook, Farjo, & Mieler, 2002). For this reason, regular screening for retinal toxicity is recommended to detect early retinopathy and discontinue the therapy. Several different techniques have been proposed so far as screening methods for the early detection of HCQ retinopathy (Browning, 2002; Marmor et al., 2002; Warner, 2001). However, most of these methods are not sensitive enough. Consequently, there is no consensus regarding the screening routine for early detection of retinopathy (Alarcon, 2002; American College of Rheumatology Ad Hoc Committee on Clinical Guidelines, 1996; Bishara & Matamoros, 1989; Easterbrook, 1999; Semmer, Lee, Harrison, & Olsen, 2008).

The purpose of the current investigation was to assess the value of central 2-10 perimetry in early detection of retinal toxicity.

## 2. Method

### 2.1 Patients

All participants gave written informed consent to participating in this investigation, which was approved by the Ethics Committee of Urmia University of Medical Sciences. Moreover, the procedures complied with the tenets of the Declaration of Helsinki and subsequent revisions. A total of 60 individuals who had normal fundoscopy were included in this cross-sectional, prospective study. The investigation was conducted on patients with rheumatoid arthritis who had been receiving HCQ for at least 6 months. All included individuals were on their medication (HCQ intake) at the time of enrollment as well. On the other hand, the exclusion criteria were: i) a history of renal or liver dysfunction, ii) use of tricyclic antidepressants, iii) a history of eye trauma, iv) amblyopia, v) a history of diseases that could alter the fundus perimetry such as gross ametropia, glaucoma, macular drusen, and other maculopathies, vi) a history of hydroxychloroquine or chloroquine intake, and vii) abnormal fundoscopic signs at the time of study.

### 2.2 Ophthalmologic Examination

All patients underwent an ophthalmologic examination including perimetry, visual acuity, and fundoscopy. An ophthalmologist examined participants using direct and indirect ophthalmoscopy and central 2-10 perimetry technique. Demographic and clinical characteristics such as age, gender, blood pressure, medical history, and dose of HCQ intake, were all retrieved from participants’ medical files. Visual field testing with automated perimetry was performed on all included subjects twice in a 6 months interval: The first perimetry was achieved at the time of enrollment whereas the second one was carried out 6 months later. All perimetric examinations were interpreted by the same ophthalmologist. The central visual field was examined with a 10-2 visual program on the Humphrey Visual Field Analyzer (Humphrey Instruments Inc, Dublin, California, USA) using red target. Individuals with systolic/diastolic blood pressures higher than 140/90 mmHg or subjects who were using antihypertensive medications were considered as hypertensives.

### 2.3 Statistical Analysis

Statistical analyses were performed by Statistical Package for the Social Sciences (SPSS ver. 19; SPSS Inc, Chicago, USA). Fisher's exact test was utilized to compare qualitative variables whereas Mann-Whitney U-test was used to compare intergroup continuous parameters. Two-sided P values less than 0.05 was considered statistically significant for all analyses.

## 3. Results

We evaluated a total of 11 men (aged 32-66 years; mean=42.73) and 49 women (aged 38-66 years; mean=40.22) in the current study. Table 1 displays the baseline characteristics in the investigated population according to the gender. None of these parameters showed a significant difference between males and females. The average duration of HCQ therapy was 5.22 years (min=8 months, max=84 months) in males and 6.31 years (min=6 months, max=120 months) in females. Anterior segment was abnormal in 9 individuals including 3 subjects with macular pigmentary changes, 4 individuals with cataract and 2 cases with dry eyes. On the other hand, 2 men and 10 women had retinal pigmented epithelium (RPE) in their posterior segments. The minimum and maximum daily HCQ doses were 45 mg and 450 mg in our population, respectively.

**Table 1:** Comparison of demographic characteristics and clinical parameters between males and females

Variable	Male (N=11)	Female (N=49)	Total (N=60)
Age [year], Mean (SD)	42.73 (7.93)	40.22 (8.14)	40.94 (7.64)
Duration of therapy [month], Mean (SD)	5.22 (2.43)	6.31 (2.86)	6.08 (2.54)
Anterior segment, N (%)			
Normal	9(81.8)	42(85.7)	51(85)
Abnormal	2(18.2)	7(14.3)	9(15)
Posterior segment, N (%)			
Normal	9 (81.8)	39 (79.6)	48 (80)
Abnormal (with RPE)	2 (18.2)	10 (20.4)	12 (20)
Hypertension, N (%)	3 (27.3)	15 (30.6)	18 (30)
BMI [Kg/m^2^], Mean (SD)	26.25 (4.09)	24.43 (3.11)	25.11 (3.64)
BCVA [logMAR], Mean (SD)	0.17 (0.26)	0.16 (0.31)	0.16 (0.28)
Daily HCQ dose [mg], Mean (SD)	161.8 (107.3)	172.2 (95.3)	168.6 (102.4)
Cumulative HCQ dose [g], Mean (SD)	235.4 (175.4)	241.5 (190.6)	239.4 (182.1)

BCVA: best corrected visual acuity; HCQ: hydroxychloroquine; logMAR: logarithm of the minimum angle of resolution scale; RPE: Retinal pigmented epithelium; mg: milligram; g: gram.

Among 60 patients who were assessed by perimetry, 48 cases (80.0%) had normal results whereas 12 subjects (28.6%) showed depressive changes after 6 months. Table 2 has summarized the results of the second visual field testing in the total population, in those who had normal fundus and in patients with normal/abnormal anterior and posterior segments. After 6 months, HCQ therapy deteriorated significantly the perimetric results of 5 (55.6%) patients (P=0.013) with abnormal anterior segment. A same trend was observed in perimetric results of 6 (50.0%) subjects with abnormal posterior segments (P=0.009). In addition, depressive changes also developed in 7 (14.6%) out of 48 patients who had normal fundus at the beginning of the study (Table 2).

**Table 2:** Results of visual field testing 6 months after the first perimetric examination.

	Perimetry		Total	P value

	Normal	Depressive		
Total population, N (%)	48 (80.0)	12 (20.0)	60	-
Patients with normal fundus, N (%)	41 (85.4)	7 (14.6)	48	-
Anterior segment, N (%)				
Normal	42(83.4)	7 (16.6)	51	0.013
Abnormal	4 (44.4)	5 (55.6)	9
Posterior segment, N (%)				
Normal	42(87.5)	6 (12.5)	48	0.009
Abnormal	6 (50.0)	6 (50.0)	12	

Table 3 compares the mean levels of daily and cumulative HCQ doses between different perimetric results of the investigated population. The daily dose of HCQ as well as the cumulative dose of hydroxychloroquine displayed statistically significant associations with perimetric results (P=0.035 and P=0.021, respectively).

**Table 3:** The mean levels of daily and cumulative HCQ doses according to perimetric results.

	Perimetry	N (%)	Mean (SD)	P value
Daily HCQ dose [mg], Mean (SD)	Depressive	12 (20)	185.7 (105.2)	0.035
	Normal	48 (80)	166.5 (93.2)	
Cumulative HCQ dose [gr], Mean (SD)	Depressive	12 (20)	257.5 (189.2)	0.021
	Normal	48 (80)	235.2 (164.8)	

HCQ: hydroxychloroquine.

## 4. Discussion

Toxic retinopathy is one of the serious side effects associated with the use of chloroquine and hydroxychloroquine. Risk factors proposed for the development of HCQ retinopathy include long duration of treatment (>5 years), high daily drug dosage (>6.5 mg/kg of lean body weight), concomitant renal or liver disease, high level of body fat, and age of older than 60 years (Bernstein, 1991; Browning, 2002; Marmor et al., 2002). Regular screening examinations are critical in early diagnosis as well as in prevention of HCQ toxicity. Nowadays, drug discontinuation is the only way to prevent ocular side effects (Marmor et al., 2002). Making the decision to cease the drug is based on diagnostic and screening examinations. However, for the most majority of patients, taking chloroquine or HCQ is the most effective way to control the underlying systemic disease. Moreover, drug cessation can lead to deteriorating of underlying disorder. In some occasions, it may also be necessary to substitute other medications such as steroids which are associated with serious systemic side effects (Yam & Kwok, 2006). Ocular screening tests have a fundamental role in early diagnosis of HCQ toxicity, and therefore should be chosen with great precision. It has been reported that central 10-2 perimetry is more sensitive than fundoscopy and color vision testing (Elder, Rahman, & McLay, 2006). In the present investigation, we aimed to evaluate the value of central 2-10 perimetry in early detection of retinal toxicity in 60 rheumatoid arthritis patients with normal fundoscopic findings.

There is a controversy about whether the dosing regimen and the total dose of Chloroquine or HCQ are associated with the risk of retinopathy (Puavilai et al., 1999). Indeed, Chloroquine retinopathy was initially observed in subjects receiving an overdose of this drug (Francois, de Rouck, Cambie, & de Laey, 1972; Grierson, 1997; M. J. Vedy, 1975). Additionally, further cases of maculopathy were described in subjects who used chloroquine prophylaxis at 100 mg daily for more than 10 years, with cumulative doses ranging around 300 g (Chovet, Vedy, Fauxpoint, & Vingtain, 1979; Metge, Rodor, Chovet, Montabone, & Llavador, 1979; Trojan, 1975; J. Vedy, Fauxpoint, Labat, Carrica, & Rivaut, 1978). It has also been shown that a cumulative dose of >700 g might be toxic, corresponding to a prophylaxis of 100 mg per day for 20 years (Chovet et al., 1979; J. Vedy, Graveline, Carrica, Rivaud, & Chanut, 1979). There are also some investigations in the literature that have shown that a cumulative dose of 185 g (Balo, Mensah, & Mihluedo, 1996) or even 140 g (Ducousso et al., 1995) should be considered as the threshold dose to avoid retinopathy. This is compatible with our findings. Hence, we suggest that, in any case, patients should be systematically screened before this cumulative dose is reached.

There is no general consensus on the definition of true hydroxychloroquine retinopathy. In the current study, those who had normal fundus and perimetric results at the beginning of the study but developed depressive changes during the follow up period were considered as HCQ retinopathic patients. According to this criterion, 21 subjects who had abnormal anterior or posterior segments were found in our population. All of these subjects were recognized using automated perimetry with red target methods. Interestingly, the mean concentration of cumulative HCQ dose was significantly higher in participants with abnormal anterior (P=0.041) and posterior (P=0.032) segments compared with subjects who had normal segments. A similar trend – with borderline P values – was also observed for the daily HCQ doses (P=0.052 for subjects with abnormal anterior segment and P=0.043 for subjects with abnormal posterior segment compared with cases with normal anterior/posterior segments).

After combining the data of 9 prospective studies, Yam and Kwok (Yam & Kwok, 2006) reported that, out of a total of 2404 patients who received HCQ therapy, only 9 patients developed retinopathy (0.4%). Moreover, in a prospective study of 526 patients, the overall incidence of irreversible hydroxychloroquine retinopathy was 0.38% (Mavrikakis et al., 2003), whereas the highest incidence of 4% occurred in a prospective study of 99 patients (Rynes, 1983). On the other hand, the incidence rate in our population was 20%. However, it seems that the majority of these patients actually are reversible premaculopathy rather than true retinopathy, when the Bernstein's (Bernstein, 1991) or Easterbrook's definitions (Easterbrook, 1993) of HCQ retinopathy were considered. Thus, more comprehensive studies, with larger sample size, longer-term follow-up and more precise techniques are mandatory to confirm HCQ retinal toxicity.
